# Free Trade in Medicine

**Published:** 1863-01

**Authors:** 


### Art. III.—FREE TRADE IN MEDICINE.

At a time when the efforts, made so largely of late, for the legislative protection of the profession in this country, have most miserably failed; when English practitioners of medicine have saddled themselves with a cumbersome regulative government, which affects the most effete red-tapism, which suffers itself to be bearded alike by charlatan and corporation, and which effects the least amount of benefit at the largest amount of cost; at a time, indeed, when the examples which, every now and then, straggle into notice of the method pursued by French courts of law in cases of quackery, and which, acting as wormwood and gall to our feelings, seem to show that the legislative enactments of our neighbours are much more fitted than our own to protect the majesty and emoluments of Physic:—at such a time it cannot fail to prove of interest to harken to a loud-complaining voice from suffering (and, if we are to believe the cry, over-governed) medicine across the Straits.

A French physician, discontented with the existing state of things within and without the profession in France, especially with the relations of Medicine to the Law, utters his complaint,* and boldly advocates the removal of all legislative restrictions on the practice of physic; averring that by such a course, coupled with a higher degree of education among authorized practitioners, quackery would be most effectually held in check, and eventually destroyed.

A Frenchman who proposes an innovation must always base it upon first principles. As the sceptic in Bishop Eurle's delightful *Microcosmography*, who "puts his foot into heresies tenderly, as a cat into water," he feels a shiver of undefinable dread at his own boldness; and instead of taking his stand upon the wants of the time, reverts at once from a revolutionary title to an inquiry into the eternal fitness of things. Hence, we are first invited to consider on what grounds of pure reason the prosecution of quacks can be justified.

It is presumed that the law, by which the proceedings of unlicensed practitioners are forbidden, must rest on the assumption that the art of medicine is of difficult acquirement, and dangerous if exercised by inexperienced hands. The design of the law, therefore, is to prevent the injury that incompetent practitioners might inflict upon their patients.

---

* *La Médecine et le Monopole.* Par le Docteur Romain Vigouroux. 8vo. pp. 45. Paris, 1862. E. Dentu.

Our author argues, however, that government regulations, of a protective character, are neither necessary nor justifiable in cases where subjects can protect themselves. He admits the danger that overhangs the victims of charlatanism, but maintains that this danger is neither unforeseen, nor, as far as the patient is concerned, unavoidable. The patient seeks the quack with his eyes open, courts the danger, and would, if he were able, utterly reject the protection.

And it is a very curious illustration of the essential difference between the French and English mind, that the inherent right of a man to choose his own doctor, whether quack or not, is defended upon the ground that there are various systems of philosophy, corresponding to the several views that may be entertained about various metaphysical problems, and that these systems of philosophy have their corresponding or analogous systems of medicine. The physician who is a rationalist founds a rationalistic school of medicine; the physician who is a spiritualist, a spiritualistic one. It is assumed, further, that the patients of each will be of corresponding mental characters; and that this correspondence will even extend from the calm domain of philosophy to the tempest-tossed region of politics. "The patients of a physician present, on the whole, a certain homogeneity of judgments and sympathies, even upon subjects foreign to medicine; and free-thinkers will seldom have recourse to the same physician, or even to the same physic, as those who are ready to submit themselves to authority." On this account Dr. Vigouroux is of opinion that every sick person is entitled to select, as his doctor, from among all mankind, the individual most homogeneous with himself; and that any restriction upon this right amounts, in fact, to a restriction upon liberty of conscience. He paints the supposed hardships of a man who cannot find the required homogeneity in the ranks of official medicine, and who cannot go beyond those ranks without complicity in an illegal action.

On this side of the Channel, the doctor's facts and arguments, up to this point, will be regarded as the merest moonshine. In this country, at least, medical schools or systems are not based upon metaphysical subtleties, but simply upon various interpretations of clinical experience; we go to the facts for our system, not to the system for our facts. And except for the famous conclusion arrived at by our most celebrated lecturer on medicine, to the effect that the disputes about contagion and non-contagion were the result of the importation, into matters medical, of the respective peculiarities of the Whig and Tory intellects, we are not aware that there is the slightest general correspondence between the medical and the general, or the political, or the religious opinions of our men of mark. Still less do their opinions on general subjects determine the nature of their connexions.

But even when regarded in this light, the demand for physic in harmony with the opinions of the patient strikes us as something at once interesting and novel. The British Medical Association is entering upon a laborious therapeutical inquiry, and we would submit that there is here an opening for careful and painstaking investigation. If Dr. Vigouroux be right, how much of the uncertainty that attends upon the action of remedies may be due to our insular carelessness, in not ascertaining the philosophical and religious creeds of the patient, as well as the symptoms of his disease. We fear there is scarcely a physician in London who would hesitate to prescribe, albeit equally ignorant and careless, as to whether his patient were a "free thinker" or a "partisan of authority." And yet, if this very circumstance be sufficient to determine a difference in the physiological action of medicines, it must probably, in some way or other, leave its mark upon the organism. It is to be feared that an inquiry into opinions about things in general would lead to tiresome delay in the consulting-room; and hence some external signs bearing upon the matter are greatly to be desired, and should be diligently sought for. Among the rising men attached to the metropolitan hospitals there must be many fully competent to undertake the necessary researches; and we trust that they will neither disregard such an opportunity of elucidating one of the hidden causes of idiosyncrasy, nor forget the immortality that would reward the inventor of an accurate ideoscope.

Returning to matters of more immediately practical import, the author thinks that the same freedom with which men can choose a religion, or an instructor for their children, should be extended to the choice of a doctor. He thinks the interests which are left to the discretion of the public are similar in kind, and even higher in degree, than the health interest that is protected. He admits that the education of the public in matters medical has yet to begin; but he thinks that the power of blundering, and the penalties which attach to blunders, would in themselves form a good foundation for it. He does not appear to see that his argument may be turned against himself. In this country there is an increasing feeling that schoolmasters should be licensed, and their competency tested, by some public body. We think, indeed, that the protection of the public against quackery is a legitimate and proper end of legislation; but, in common with Dr. Vigouroux, we doubt if any real protection be afforded under the present system.

The protection of the profession against irregular competition Dr. Vigouroux dismisses at once, as a claim not to be entertained for a moment. He points out, with perfect truth, that many other callings require a prolonged and expensive education, and usually entail a weary period of waiting for employment, but that the professors of these callings receive no protection from the State. He points out, further, that the protected industries are only those that are too inherently weak to stand alone; and he refers to the doctrines of free trade as containing the only true philosophy of such matters. The next chapter of the pamphlet is devoted to a description of the processes against quackery in a French court of law. There are many Englishmen who would be glad to see analogous processes in our own courts, and we recommend this portion of *La Médecine et le Monopole* to their careful perusal. The account given may be summed up by saying, that the person arraigned has opportunity of producing in open court witnesses ready to testify to marvellous cures; and that he thus gains a notoriety cheap at the fifteen francs of penalty. Moreover, where medical practitioners or corporations are the persons to set the law in motion, any failure of the prosecution becomes the victory of the charlatan in his lawsuit with the faculty.

It is unnecessary to follow Dr. Vigouroux step by step through his pamphlet. Suffice it to say, that he exhibits the inefficiency of the existing law and the necessity for an alteration, points out that a change in the direction of greater severity desired by some would fail by reason of the violence it would do to public opinion, and would inevitably pave the way to a removal of all restrictions; and traces the causes of quackery, or the support it receives from the public, to two chief causes, of which the first is popular ignorance on all matters of natural science, the next, the injury done to the medical profession as a body by the existence of the "*officier de santé*," who with scanty knowledge, imperfect education, and little scrupulousness, commits errors of diagnosis, of treatment, and of general conduct, the reproach of which is shared with him by men who could not have fallen into them. The public, little versed in the distinctions between different kinds of medical qualification, condemn the whole body for the failings of a few, and estimate the physician by their experience of the *officier*. Dr. Vigouroux states that the latter grade is defended on the ground of a presumed necessity to provide a cheap doctor for the poor; and that this necessity has its origin, so far as it is real, in the laws which totally forbid the practice of the unqualified. He thinks the poor, if they must have an inferior article, had better have it unticketed, and that the *officiers* would be advantageously replaced by practitioners wholly unqualified (*les médecins non grades*), whose claims to confidence would rest upon their own conduct and apparent skill, rather than upon a cheap diploma, testifying to an inferior and superficial education. He advocates, therefore, the abolition of the restrictive laws and of the grade *officier*, and the adoption of a curriculum and examination which would make the degree of a physician unattainable, save by men of very considerable ability and knowledge.

At the present time, when it is reported that the Medical Council have prepared or are preparing a bill to punish and prevent illegal practice, and when they would be supported in doing so by the voices of many practitioners, the appearance of this little pamphlet is singularly opportune. We are strongly impressed with the belief that the suppression of quackery must be attempted by striking at its causes; and that the imprisonment or other punishment of offenders will leave the evil where it was, and, at most, will only seclude a single professor for a very brief period. A prison will no more reform a quack than it will a pickpocket; and public opinion would not tolerate any severe punishment, any that would really exert a deterring influence. Indeed, in this country, as in France, any legal penalty that could be enforced would probably be courted as a sort of cheap martyrdom and means of notoriety, and would be found in no way to diminish, even if it did not augment, the mischief.

The cause of quackery, we apprehend, is, in about one case in ten, the disappointment of the unreasonable expectations of the sick. A patient desires something that is beyond the reach of our art, the cure of malignant disease, for example; or else wishes to be well without submission to the necessary discipline or treatment. In either case, the surgeon being honest and speaking the truth, a quack is applied to, who is dishonest and does not object to tell lies for a consideration, or who is probably too ignorant even to know when he is doing so. In the case of a patient who refuses to submit to treatment, it is quite conceivable that serious mischief may be the result. The disease may be one (such as strangulated hernia or acute glaucoma) in which time is of infinite value, in which the performance of an operation within a certain number of hours is absolutely necessary in order to preserve life or to restore healthy action; and the patient must then suffer the consequences of his folly. But such instances are very, very rare. In the great issues of life men usually take care to ascertain the value of the advice they follow. As a matter of fact, the unreasonable expectations that take people to quacks are almost confined to incurable or chronic ailments, in which the mischief done by want of treatment is comparatively small; only just enough, perhaps, to convince the sufferer of its reality, and to lead him back, in penitent submission, to the hands of the authorized practitioner. The remedy, we think, can only be sought in the diffusion of knowledge, and especially knowledge of the elements of physiology and of sanitary science. As these become more and more spread abroad among the community, the bases of medical science and the nature of the difference between the physician and the quack will become daily more and more apparent.

The remaining nine-tenths of existing quackery can be traced, we greatly fear, to the disappointment of reasonable expectations; and we are well assured that quackery will flourish in any district, precisely in the ratio in which the local surgeons provide good grounds for such disappointment. If the doctor be either ignorant or careless, a certain proportion of his patients will remain on hand unrelieved, so as to lower the public estimate of his ability, and to impair that confidence in him that is so needful to his success. Of this proportion it is not too much to say that some will probably be injured by treatment, and will improve when it is discontinued. If these should by any chance go to a quack or a homœopath, and receive some inert medicine, lo! a wonderful cure has been effected.

If the doctor be rapacious, and bent only upon extorting the uttermost farthing from his patients, he will, usually, be ignorant and careless as well. But greed will add terribly to the effect of the other disqualifications, and will help the local quacks immensely. People can get rid of them; but they are afraid to let such a doctor gain his footing in their houses. So they go to a druggist, or to a seller of worm powders, or to anybody who will, they think, help them to stave off the evil beginnings of a bill, or perhaps to avoid them altogether.

And why should doctors be ignorant, or careless, or greedy? Alas! we are all patients in the same hospital, suffering all alike from some degree of these and many other infirmities, and no hope is held out to us of any but a gradual cure. Still it is worth while to see whether, over and above the necessary frailty of human nature, there may not be special causes admitting of removal. We think such causes must be sought, almost wholly, in defective education, and especially in defective preliminary education. The student who goes to a hospital without previous mental and moral training, has neither power to acquire and arrange knowledge, nor perception of the importance of doing so. He is apt to look on his profession and his acquirements as matters that are for his own sole benefit, and not as involving very heavy responsibilities in the way of duties towards his fellow-creatures. From this point of view he attends to what he likes, and neglects what he likes, as a matter of inclination or of indolence, rather than of conscience; and when time calls him to take his place in life, he still recognises no claim in his profession, either to know or to do, which he may not postpone to his own profit or convenience. It would be hard, and perhaps unjust, to say that the heads of the profession in London have and perceive a direct interest in keeping down the standard of skill and knowledge among the many; but the fatal facilities that have been given for the entrance of uneducated men into our ranks, the indecent mutiny of the Council of the College of Surgeons against the Medical Council, and the absence of any provision that could lead to a better state of things, all go near to justify a belief that such an interest has been both perceived and acted upon. The men who frame curricula are practically acquainted with the duties of a medical practitioner; and they cannot be ignorant how little the regulations they have devised are, in themselves, calculated to produce licentiates who will discharge those duties in a creditable manner.

Of the ignorance and carelessness that depend upon moral torpor, upon the absence of a sense of duty in connexion with professional study and practice, greed is an invariable concomitant. The routine of medical work is so unpleasant, in many respects, that it can only be thoroughly accomplished under the influence of scientific enthusiasm, sense of duty, or desire of gain. Perhaps it is best accomplished when they are combined; but the absence of one, and *a fortiori* of two of them, must either receive compensation from excessive development of the third, or must involve an absolute incapacity to discharge the ordinary duties of a practitioner. Some men find out this incapacity for themselves; others receive tacit information on the subject from the public. But there are many who have no love of science whatever, and who have but a limited sense of duty, who still go through the outward forms of medical attendance upon the sick in a punctual and methodical manner. From such men there proceeds an atmosphere eminently favourable to the development of quackery.

There is, however, a kind of carelessness, very hurtful in its influence upon the sick, and ten times more hurtful in its reaction upon those who practise it, and through them upon the profession, which proceeds from very different causes, and chiefly from the numerical inadequacy of the regulation "staff" of a hospital or dispensary. It is chiefly to the out-patient department that this observation will apply, and we will illustrate it by the practice of one of the best hospitals in London.

At the institution in question, the charge of about nine thousand out-patients yearly devolves upon three assistant-physicians. These gentlemen attend from one o'clock until about half-past two, each of them twice a week; and, except by special order, no patient is allowed to come before them on both these days. A few do so, and a few are allowed to come once a fortnight, but as a rule, one attendance weekly is the practice. A large proportion of these out-patients suffer from complicated chronic disease, and many of them are so poor that their improvement is hindered by want of comforts or even of necessaries. We shall be within the mark if we allow six visits to the hospital for each person. It follows that the assistant-physician, in an hour and a half, is called upon to prescribe for about 173 persons, of whom 29 will be new cases.

Of course it will occasionally happen that the practised eye of the physician detects, in a moment, something that does not make itself apparent to less skilled observers. The patient will be detained, carefully examined by the doctor and by the students, the disease accurately diagnosed, the treatment skilfully directed. But what is the rule? The first glance, or the most salient symptom, is taken as the basis of a prescription; and to every patient who can say "better," the words "go on the same" are repeated with a rapidity worthy of Charles Mathews. We well remember that one of the assistant physicians was accustomed to devote to the instruction of the students as much time as they could save him. Before his arrival, and even in his presence, they used to call in as many patients as their own persuasions and the persuasions of the hall-porter could induce to forego their right to an interview with the great man, and to despatch them with marvellous rapidity. And then, at the close of this proceeding, the doctor would turn his chair to the fire, throw the contents of his snuff-box, for easier access, upon a sheet of paper, say that he had ten minutes or twenty minutes, as the case might be, ask what he should talk about, and pour forth such stores of wit, and wisdom, and knowledge, from the exuberance of his fertile fancy, his magnificent intellect, his unequalled memory, that those hasty and extempore lessons can never fade from the minds of his hearers, their principles never be lost, their precepts never be forgotten. The voice that uttered them is now silent, the speaker is added to the number of those whose lives, worn out by incessant physical and mental toil, have been spent in the mere attainment of an eminence, the rewards of which were still in the future; and now, in looking back upon the past, partiality itself could not deny the abandonment of duty involved in the custom we have described.

It would be absurd of course, to test any universal system by the powers of a great and exceptional genius; and it is manifest that the number of persons prescribed for under the conditions detailed above, must not only preclude correctness of diagnosis in very many instances, but must even lead continually to errors and oversights of the most important kind. Even if this were not the case, the habit of hasty and superficial examination would be formed, and this habit, hurtful even to the physician himself, would be doubly hurtful as an example to half-instructed students. There are not wanting young men who aim at rapidity of diagnosis, and who simply cultivate a habit of jumping at inaccurate conclusions. We have lately seen an instance in which a practitioner of this type, called to a poor woman who was vomiting, rested upon that symptom a diagnosis of *dyspepsia*, and a treatment by what is called "Mist: Sodæ Co." He did not discover that the vomiting was stercoraceous, nor anticipate the death of the patient, which occurred (from intestinal strangulation) about six hours after the visit. Sir Henry Halford, who was accustomed to pay twenty visits in an hour, and Sir Benjamin Brodie, whose marvellous rapidity of perception needs no description here, were the models whose practice this gentleman professed to follow. We have seen, also, a case of pain (said to be obscure) attending certain movements of the hip and thigh, and variously treated during some months, under various hypotheses as to its nature, but always without benefit, by some three or four of the leading men of London. At last it occurred to a very undistinguished practitioner to examine the seat of pain, with the result of discovering a simple clue to the apparent mystery. None of the physicians or surgeons who had prescribed in vain had bethought them of this method of inquiry.

Now, carelessness such as this disappoints reasonable expectations, and by doing so is a fertile source of quackery. People can tell when the great hospital surgeon desires only to get his guineas, and to clear his room as fast as possible ; they can tell when the humble general practitioner does not know what is the matter, and does not want to know, and does not care whether they are better or worse, except in so far as the former condition may curtail his charges, or the latter diminish his reputation. Is it not obviously true that the prosperity of quackery is the measure of want of confidence in legitimate medicine ? Is it not obviously true that a man who understands his business thoroughly, and who is in earnest about it, cannot fail to command the confidence of all who are brought into contact with him ? We think that both these propositions are undeniable; and we say to those of our brethren who hate quackery as a crime and a nuisance, and who really desire its destruction, that the way thereto is to remember that every professional opinion and act tends to the elevation or to the disgrace of legitimate medicine, and thereby to the abolition or the encouragement of quackery.

Every erroneous judgment, every instance of malpractice, is a point ceded to the enemy; and while we remember that errors cannot be excluded from human effort, and therefore judge others charitably, let us remember also how many of our own errors depend upon ignorance about matters that we ought to know, or upon carelessness about points that we ought to observe. For the present generation individual care and watchfulness will do much, and for those who will succeed us careful preliminary education will do more. When avoidable errors are in great measure excluded, when we are prepared to satisfy the reasonable expectations of our patients, the demand for quacks will have reached a vanishing point, and the trade that is now so profitable will cease to be worth pursuing. Penal legislation against quackery would produce either a *tracasserie* or a persecution; the first futile, the second unbearable.

In proportion, however, as we elevate our standard of education, shall we be justified in seeking from the Government protection against false pretenders to the medical character. We would leave the corn cutter, the worm doctor, the bone setter, the "botanist," the Lady Bountiful, the country parson, and the country parson's wife, at liberty to physic, all and sundry, either for hire or for charity, as many as choose to resort to them; but we would consign to the oakum-yard or the treadmill, without a shadow of compunction, all persons, who not being duly qualified members of the medical profession, used any title or committed any act calculated to lead to the belief that they were so qualified.

---

